# Quantifying Patient-Specific *in vivo* Coronary Plaque Material Properties for Accurate Stress/Strain Calculations: An IVUS-Based Multi-Patient Study

**DOI:** 10.3389/fphys.2021.721195

**Published:** 2021-10-25

**Authors:** Liang Wang, Jian Zhu, Akiko Maehara, Rui Lv, Yangyang Qu, Xiaoguo Zhang, Xiaoya Guo, Kristen L. Billiar, Lijuan Chen, Genshan Ma, Gary S. Mintz, Dalin Tang

**Affiliations:** ^1^School of Biological Science and Medical Engineering, Southeast University, Nanjing, China; ^2^Department of Cardiology, Zhongda Hospital, Southeast University, Nanjing, China; ^3^The Cardiovascular Research Foundation, Columbia University, New York, NY, United States; ^4^School of Science, Nanjing University of Posts and Telecommunications, Nanjing, China; ^5^Department of Biomedical Engineering, Worcester Polytechnic Institute, Worcester, MA, United States; ^6^Mathematical Sciences Department, Worcester Polytechnic Institute, Worcester, MA, United States

**Keywords:** coronary plaque, *in vivo* material properties, vulnerable plaque, artery material properties, plaque stress, artery model

## Abstract

**Introduction:** Mechanical forces are closely associated with plaque progression and rupture. Precise quantifications of biomechanical conditions using *in vivo* image-based computational models depend heavily on the accurate estimation of patient-specific plaque mechanical properties. Currently, mechanical experiments are commonly performed on *ex vivo* cardiovascular tissues to determine plaque material properties. Patient-specific *in vivo* coronary material properties are scarce in the existing literature.

**Methods:** *In vivo* Cine intravascular ultrasound and virtual histology intravascular ultrasound (IVUS) slices were acquired at 20 plaque sites from 13 patients. A three-dimensional thin-slice structure-only model was constructed for each slice to obtain patient-specific *in vivo* material parameter values following an iterative scheme. Effective Young's modulus (YM) was calculated to indicate plaque stiffness for easy comparison purposes. IVUS-based 3D thin-slice models using *in vivo* and *ex vivo* material properties were constructed to investigate their impacts on plaque wall stress/strain (PWS/PWSn) calculations.

**Results:** The average YM values in the axial and circumferential directions for the 20 plaque slices were 599.5 and 1,042.8 kPa, respectively, 36.1% lower than those from published *ex vivo* data. The YM values in the circumferential direction of the softest and stiffest plaques were 103.4 and 2,317.3 kPa, respectively. The relative difference of mean PWSn on lumen using the *in vivo* and *ex vivo* material properties could be as high as 431%, while the relative difference of mean PWS was much lower, about 3.07% on average.

**Conclusion:** There is a large inter-patient and intra-patient variability in the *in vivo* plaque material properties. *In vivo* material properties have a great impact on plaque stress/strain calculations. *In vivo* plaque material properties have a greater impact on strain calculations. Large-scale-patient studies are needed to further verify our findings.

## Introduction

Cardiovascular diseases, such as heart attack and stroke, are the number 1 cause of death globally, and killed more than 17.7 million people in 2017 (GBD 2017 Causes of Death Collaborators, 2018). The underlying cause for cardiovascular diseases (CVDs) is atherosclerotic plaque progression and rupture, which involve complex pathophysiological, biochemical, and biomechanical factors among others (Stary et al., 1995; Malek et al., 1999; Virmani et al., 2000; Yang et al., 2009). To investigate the biomechanical mechanisms governing these plaque behaviors, image-based computational models have been developed to simulate biomechanical conditions in patient-specific settings for better disease diagnosis, treatment, and prognosis (Tang et al., 2004; Samady et al., 2011; Stone et al., 2012; Gijsen et al., 2015). However, precise quantifications of biomechanical conditions depend heavily on accurate material properties of patient-specific plaque tissues (Akyildiz et al., 2014).

Extensive efforts have been made to determine the material properties of cardiovascular tissues (Fung, 1993; Holzapfel et al., 2000; Maher et al., 2009; Walsh et al., 2014). Fung et al. conducted a uniaxial loading test on specimens from a healthy canine aortic tree and observed that the stress-stretch ratio curve of the cardiovascular tissue was typically in exponential form. Therefore, a Fung-type-material model was proposed to describe the material properties for these tissues (Fung, 1993). To study the mechanical properties of cardiovascular tissues with atherosclerotic disease, Holzapfel et al. (2004) examined plaque tissues in iliac artery. Experimental data indicated that tissue properties were highly nonlinear and anisotropic. An anisotropic Mooney–Rivlin material model was introduced to describe the mechanical properties of the atherosclerotic plaques (Holzapfel et al., 2000). Furthermore, more detailed information on layer-specific and component-specific material properties of carotid plaque were also documented, and a large inter-specimen variation was reported (Teng et al., 2014; Hoffman et al., 2017). Even though considerable experimental data have accumulated based on *ex vivo* tissues, it is still desirable to use patient-specific *in vivo* material properties in computational modeling for better accuracies in disease management.

To overcome this limitation, attempts have been made to determine patient-specific *in vivo* plaque material properties. Based on Cine-magnetic resonance imaging, Wang et al. (2017) have quantified plaque material properties in carotid arteries for 16 patients. However, existing literature on *in vivo* coronary atherosclerotic plaque material properties is scarce. Maso Talou et al. (2018) have introduced a data assimilation scheme to estimate the isotropic material properties of coronary vessel wall using intravascular ultrasound (IVUS) images. Recently, our group has proposed a three-dimensional (3D) thin-slice structure-only model to determine the material properties of coronary plaque based on *in vivo* Cine IVUS images (Guo et al., 2017), but inter-patient variation in the material properties has not been reported, and its impact on biomechanical conditions has not been assessed on a multi-patient scale.

In this article, *in vivo* Cine IVUS and virtual histology IVUS (VH-IVUS) data of atherosclerotic plaques were acquired from 13 patients. An iterative procedure was performed to obtain patient-specific *in vivo* material parameter values for each VH-IVUS slice by matching the Cine IVUS data under both systolic and diastolic pressure conditions. Three-dimensional thin-slice structure-only models were used in the iterative procedures to save model construction time. A comparison of the stress/strain conditions using *in vivo* material properties and previously published *ex vivo* material properties was performed to quantify their impact on biomechanical conditions.

## Materials and Methods

### Data Acquisition

**Figure 1:**
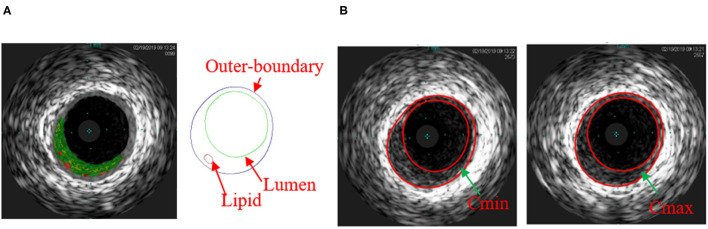
**(A)** Virtual histology intravascular ultrasound (VH-IVUS) slice and its segmented contour showing the plaque components of one sample plaque; **(B)** matched IVUS slices with minimum lumen circumference (Cmin) and maximum lumen circumference (Cmax). Colors used in VH-IVUS: red, lipid rich necrotic core; dark green, fibrous; light green, fibro-fatty.

*In vivo* intravascular ultrasound images were acquired from 13 patients (4 males, mean age: 65.4) with atherosclerotic coronary artery disease at Zhongda Hospital, Southeast University (Nanjing, China), and an informed consent was obtained. This study was part of a larger clinical study approved by the Medical Ethics Committee of Southeast University, and registered at ClinicalTrials.gov (NCT number: NCT03195621). Patient demographical and clinical information (Dodge et al., 1992) are provided in Table 1. Data acquisition procedures were described in Guo et al. (2017) and briefly summarized here. Grayscale IVUS images were obtained by scanning cross-sections of coronary plaques using a 20-MHz, 2.9-French catheter (Eagle Eye Platinum; Volcano Corp., Rancho Cordova, CA, United States). VH-IVUS images were created to provide the maps of four different types of plaque tissues: lipid-rich necrotic core (lipid in short), calcification, fibrous, and fibro-fatty tissues (see Figure 1A) (Nair et al., 2002). During IVUS image acquisition, a catheter was paused at one or two plaque sites for about 2 s for each patient to obtain the Cine IVUS images. When the catheter was paused, Cine IVUS slices recorded plaque cross-section movement over the cardiac cycle, and one corresponding VH-IVUS slice was acquired at the site to provide the plaque component information for model construction. In total, 20 VH-IVUS slices from 20 plaque sites were paused for the 13 patients. In-house atherosclerotic plaque imaging analysis (APIA) software written in MATLAB was used to automatically generate contour plots of lumen, vessel out-boundary, and plaque components, including lipid and calcification (Yang et al., 2009). The segmentation of Cine IVUS images was performed using the method similar to Giannogloua et al. (2007). Lumen circumferences for all the IVUS images in one cardiac cycle were calculated, and two IVUS slices with minimum and maximum lumen circumferences (denoted as Cmin and Cmax, respectively) were selected to represent plaque geometries under diastolic and systolic pressure conditions, respectively. Lumen circumference and arm cuff pressure data are also given in Table 1. Figure 1 shows a VH-IVUS slice, its segmented contours, and corresponding IVUS slices with Cmin and Cmax corresponding to diastolic and systolic pressures from one sample plaque site.

**Table 1:** Patient demographical and vessel segment information.

**Patient ID**	**Age**	**M/F**	**BP (mmHg)**	**Diseased artery and stenosis severity by diameter**	**Clinical history**	**Treatment and Medication**	**Site no**.	**Cmin (cm)**	**Cmax (cm)**
P1	53	M	64-107	Middle LAD 50% stenosis, near 2^nd^ diagonal branch	HT	OMT	Site 1	0.8508	0.8859
							Site 2	0.8672	0.9148
P2	63	M	86-138	Proximal to middle LAD 47% stenosis, near the 1^st^ septal branch	HT	PCI with OMT	Site 1	0.8115	0.8257
P3	58	F	91-148	Proximal LCX with 53% stenosis, distal to LM ostium	Previous CAD, HT, DM	OMT	Site 1	1.0646	1.1189
							Site 2	0.9725	1.0125
P4	70	F	94-162	Middle RCA with 44% stenosis, near acute marginal branch	HT	PCI with OMT	Site 1	1.1064	1.1411
							Site 2	1.1269	1.1724
P5	73	F	73-143	Middle RCA with 50% stenosis, proximal to acute marginal branch	HT, Stroke	PCI with OMT	Site 1	1.1846	1.2604
P6	52	F	70-126	Middle LCX with 45% stenosis, distal to the 1^st^ obtuse marginal branch	HT	PCI with OMT	Site 1	1.1371	1.1893
P7	79	F	59-124	Middle LAD with 65% stenosis, around ostium of 2^nd^ diagonal branch	HT	PCI with OMT	Site 1	1.0033	1.0312
P8	53	M	95-146	Middle LAD with 42% stenosis, distal to 1st septal branch	HT	OMT	Site 1	1.1912	1.2521
P9	72	F	81-144	Distal LCX with 55% stenosis, distal to 2^nd^ marginal branch	HT	PCI with OMT	Site 1	0.5790	0.5999
							Site 2	0.9648	1.0035
P10	64	F	68-118	Middle LAD with 60% stenosis, near 2^nd^ diagonal branch	No	OMT	Site 1	0.8913	0.9432
							Site 2	0.6882	0.7014
P11	76	F	69-137	Middle LAD with 45% stenosis, between 1^st^ and 2^nd^ septal branch;	No	OMT	Site 1	0.9500	0.9825
							Site 2	1.3395	1.4178
P12	79	F	80-160	Middle LAD with 54% stenosis, near 2^nd^ septal branch	HT	OMT	Site 1	0.8131	0.8683
P13	58	M	70-130	Proximal to middle RCA with 40% stenosis, proximal to acute marginal branch	Previous CAD	OMT	Site 1	0.9586	0.9988
							Site 2	1.1952	1.2453

*BP, blood pressure; LAD, left anterior descending; LCX, left circumflex artery; RCA, right coronary artery; LM, left main; PCI, percutaneous coronary intervention; OMT, optimal medical therapy (normally aspirin and other medications, such as statins, beta blockers, and nitroglycerin, if needed, for specific patient); Cmin, minimum lumen circumference; Cmax, maximum lumen circumference*.

### Three-Dimensional Thin-Slice Structure-Only Model

**Figure 2:**
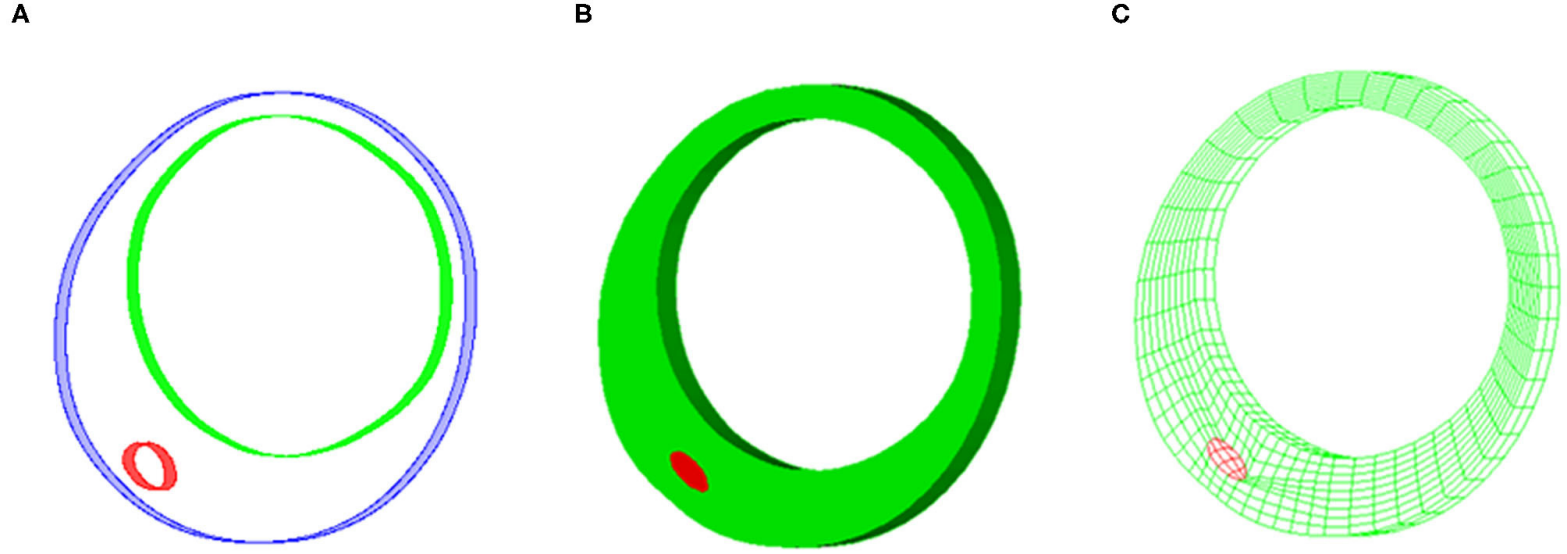
Sample plaque showing reconstructed plaque geometry and finite element mesh for three-dimensional (3D) thin-slice structure-only model. **(A)** Reconstructed 3D thin-slice plaque geometry. **(B)** Reconstructed plaque volume. **(C)** Finite element mesh.

A 3D thin-slice structure-only model was constructed for each VH-IVUS slice to quantify its patient-specific *in vivo* material properties (Huang et al., 2016). A thickness of 0.05 cm was added to the VH-IVUS slice to reconstruct the 3D plaque geometry (see Figures 2A,B). The governing equations of the structure-only model include equation of motion, the nonlinear Cauchy-Green strain-displacement relation, and material model of plaque tissues (Huang et al., 2016). Pulsating pressure conditions were prescribed at the luminal surface to mimic plaque movement.

### The Mooney–Rivlin Material Model

The coronary vessel, fibrous, and fibro-fatty tissues were treated as the same hyperelastic, anisotropic, nearly incompressible, and homogeneous plaque tissues, as prior experimental study indicated that these tissues had similar mechanical properties (Teng et al., 2014). The anisotropic Mooney–Rivlin material model with the following strain energy density function was used to describe their mechanical properties (Holzapfel et al., 2000; Yang et al., 2009):


(1) $$\begin{array}{l} W=W_{iso}+W_{aniso} \end{array}$$

(2) $$\begin{array}{l} W_{iso}=c_{1}\left(I_{1}-3\right)+c_{2}\left(I_{2}-3\right)\\ \;+D_{1}\left[\exp\left(D_{2}\left(I_{1}-3\right)\right)-1\right] \end{array}$$

(3) $$\begin{array}{l} W_{aniso}=\frac{K_{1}}{K_{2}}\left\{\exp\left[K_{2}{\left(I_{4}-1\right)}^{2}\right]-1\right\} \end{array}$$

where I_1_ = ∑C_ii_ and I_2_ = ½ [${\text{I}}_{1}^{2}$ – *C*_*ij*_*C*_*ij*_] are the first and second invariants of right Cauchy–Green deformation tensor C defined as C = [C_ij_] = X^T^X, X = [X_ij_] = [∂x_i_/∂a_j_]; (x_i_) is current position; (a_i_) is original position; I_4_ = C_ij_(n_c_)_i_(n_c_)_j_; n_c_ is the unit vector in the circumferential direction of the vessel. c_1_, c_2_, D_1_, D_2_, K_1_, and K_2_ are material parameters whose values were to be determined using *in vivo* Cine IVUS data following an iterative scheme (see more details in Iterative Scheme to Determine *in vivo* Plaque Material Parameters Values section). The material constants from *ex vivo* biaxial loading test: c_1_ = −1312.9 kPa, c_2_ = 114.7 kPa, D_1_ = 629.7 kPa, D_2_ = 2, K_1_ = 35.9 kPa, and K_2_ = 23.5, were adopted as the initial guesses to determine *in vivo* plaque material constants (Kural et al., 2012).

Plaque components (lipid and calcification) were assumed to be hyperelasic, isotropic, and nearly incompressible, and their strain energy density functions were used the form given by Eq. (2). The values of material parameters were fixed for all the slices. For lipid, we used c_1_ = 0.5 kPa, c_2_ = 0, D_1_ = 0.5 kPa, and D_2_ = 1.5; for calcification, we used c_1_ = 920 kPa, c_2_ = 0, D_1_ = 360 kPa, and D_2_ = 2. The material parameter values for calcification were chosen so that its effective YM was 10 times as much as that of the vessel wall using *ex vivo* biaxial material testing data we published earlier (Kural et al., 2012), i.e., effective YM of calcification = 10 ^*^ (YMa + YMc)/2. YMa and YMc are effective YMs of *ex vivo* plaque material in the axial and circumferential directions, respectively.

### Iterative Scheme to Determine *in vivo* Plaque Material Parameter Values

**Figure 3:**
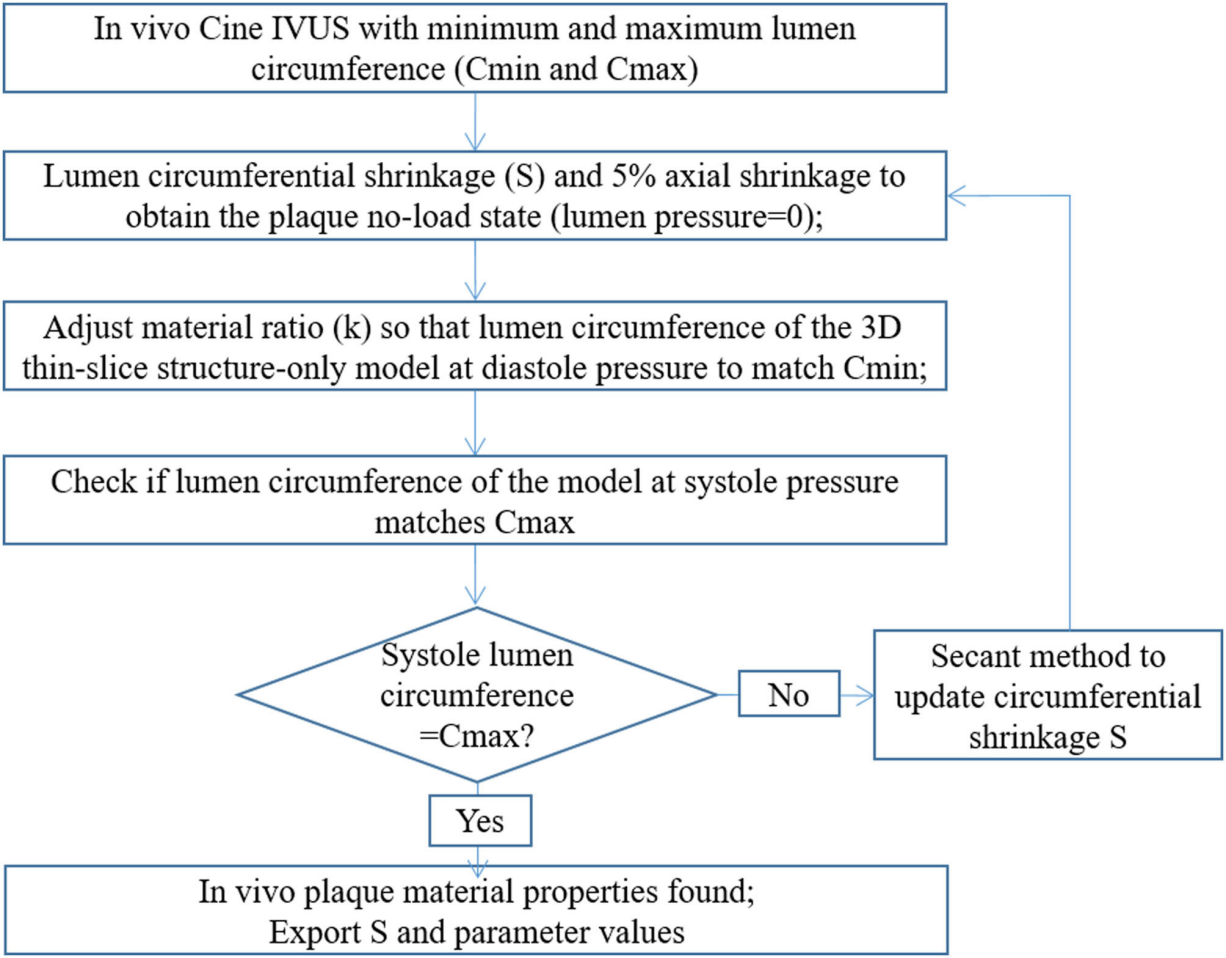
Flowchart of the iterative scheme to quantify *in vivo* plaque material properties.

Since the IVUS images were acquired under *in vivo* conditions with physiological pressure on the luminal surface and axial stretch, a pre-shrink-stretch process was performed to obtain no-load geometry of the plaque (corresponding to zero-pressure condition) as the initial geometry to start the computational simulation. More specifically, *in vivo* plaque geometry was shrunk circumferentially and axially to reach the no-load geometry. Axial shrinkage rate was fixed at 5% in our models, because atherosclerotic vessels were stiff (Guo et al., 2017). Patient-specific circumferential shrinkage rates, along with *in vivo* plaque material properties, were to be determined using the iterative scheme shown in Figure 3.

In theory, since we have only two data points to work with (minimum and maximum lumen circumferences corresponding to diastolic and systolic pressures), only two quantities can be determined. One quantity to be determined is lumen shrinkage rate S whose initial value was set to 2% in the iterative process. Once lumen shrinkage rate was set, the outer-boundary shrinkage rate was calculated using the conservation of the vessel volume. Now, only one condition is left for us to determine, the second quantity. This quantity was chosen to be a material ratio k used to determine the *in vivo* plaque material properties. More specifically, the *in vivo* plaque material parameter values were adjusted proportionally to those of the *ex vivo* material parameters, that is, c_1_ = k^*^(−1,312.9) kPa, c_2_ = k^*^114.7 kPa, D_1_ = k^*^629.7 kPa, K_1_= k^*^35.9 kPa, while D_2_ = 2 and K_2_ = 23.5 were kept fixed. In the iterative process, lumen shrinkage rate and material ratio were adjusted iteratively until the lumen circumferences from our thin-slice structure-only model matched those of Cine IVUS at both diastolic and systolic pressures (relative error < 1%).

For easy comparison, effective YM was calculated to indicate the plaque stiffness for each material curve. Effective YM (will be referred to as YM for simplicity) was defined as the slope of the proportional function that best fits a given material stress-stretch ratio curve on the stretch ratio interval [1.0 1.1] (Wang et al., 2017).

### Solution Method and Model Comparison

Finite element mesh was generated using a component-fitting mesh generation technique described in Yang et al. (2009). Figure 2C shows a sample slice with finite element mesh. The 3D thin-slice structure-only models were solved with the commercial finite element software ADINA (Adina R & D Inc., Watertown, MA, United States) following established procedures (Bathe, 2002; Yang et al., 2009). A mesh analysis was performed by refining mesh density by 10% until changes in solutions became <2%. Three cardiac cycles were simulated in our computational models, and the solution in the last period was used. In order to investigate the impact of *in vivo* material properties on calculations of biomechanical conditions, another model with *ex vivo* material properties was constructed for each plaque, and average values of maximum principal stress/strain on lumen [denoted as plaque wall stress/strain (PWS/PWSn)] were compared.

## Results

### Patient-Specific *in vivo* Plaque Material Properties

**Figure 4:**
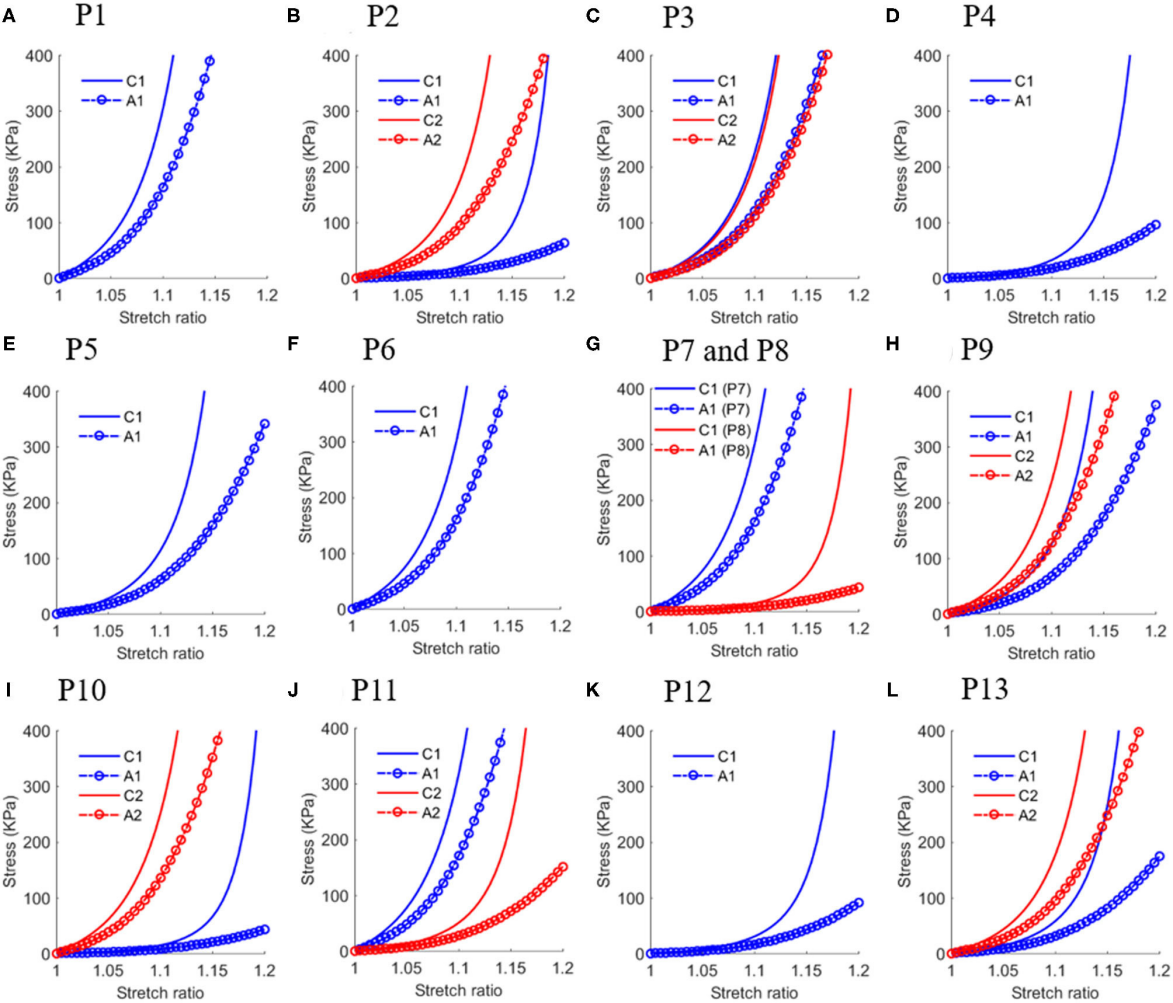
Stress-stretch ratio curves in circumferential and axial directions of *in vivo* plaque material properties with each sub-figure **(A–L)** plotted the curves for one patient except **(G)** plotted curves for P7 and P8 to save space. Abbreviation: C1 - Circumferential direction at Site 1; A1 - Axial direction at Site 1; C1 - Circumferential direction at Site 2; A2 - Axial direction at Site 2.

The *in vivo* material properties were quantified for 20 slices from the 13 patients. The values of material parameters and lumen circumferential shrinkage rate are listed in Table 2. Corresponding stress-stretch ratio curves in both circumferential and axial directions are shown in Figure 4.

**Table 2:** Values of material parameters, circumferential shrinkage rates, and Young's modulus (YM) in both directions of the 20 slices.

**Material parameters**		**S (%)**	**C_1_ (kPa)**	**C_2_ (kPa)**	**D_1_ (kPa)**	**K1 (kPa)**	**YMa (kPa)**	**YMc (kPa)**
*Ex vivo* material		–	−1,312.9	114.7	629.7	35.9	937.8	1,631.2
P1	Site 1	7.38	−340.0	29.7	163.1	9.3	242.9	422.4
	Site 2	2.25	−1,037.6	90.7	497.7	28.4	741.2	1,289.2
P2	Site 1	2.00	−1,210.3	105.7	508.5	33.1	864.5	1,503.7
P3	Site 1	17.48	−122.0	10.7	58.5	3.3	87.1	151.5
	Site 2	4.46	−1,029.0	89.9	493.5	28.1	735.0	1,278.4
P4	Site 1	2.03	−1,339.2	117.0	642.3	36.6	956.6	1,663.8
	Site 2	4.19	−1,216.1	106.2	583.3	33.3	868.7	1,511.0
P5	Site 1	13.34	−187.6	16.4	90.0	5.1	134.0	233.0
P6	Site 1	4.40	−667.6	58.3	320.2	18.3	476.8	829.4
P7	Site 1	2.00	−1,757.8	153.6	843.1	48.1	1,255.6	2,183.9
P8	Site 1	20.83	−83.2	7.3	39.9	2.3	**59.4**	**103.4**
P9	Site 1	3.49	−672.3	58.7	322.5	18.4	480.2	835.3
	Site 2	3.90	−1,388.2	121.3	665.8	38.0	991.6	1,724.7
**P10**	Site 1	17.63	−84.5	7.4	40.5	2.3	60.4	105.0
	Site 2	2.00	−1,477.6	129.1	708.7	40.4	1,055.4	1,835.7
P11	Site 1	1.64	−1,865.1	162.9	894.6	51.0	**1,332.2**	**2,317.3**
	Site 2	11.52	−295.9	25.9	141.9	8.1	211.4	367.7
P12	Site 1	15.52	−177.9	15.5	85.3	4.9	127.1	221.0
P13	Site 1	3.86	−892.6	78.0	428.1	24.4	637.6	1,109.0
	Site 2	3.86	−941.5	82.2	451.5	25.7	672.5	1,169.7
Mean		7.19	−839.3	73.3	402.5	22.9	599.5	1,042.8

*S, lumen shrinkage rate; YMc, YM value in circumferential direction; YMa, YM value in axial direction. The minimum and maximum values are in bold*.

The YM values for each slice at both directions are also given in Table 2. The average YM values in the circumferential and axial directions (denoted as YMa and YMc, respectively) for the 20 plaque samples are 599.5 and 1,042.8 kPa, respectively. This is 63.9% as stiff as the *ex vivo* material. The softest plaque had a YMc value of 103.4 kPa, while the stiffest one had YMc2317.3 kPa, 21.4 times greater than that of the softest one. This demonstrates that there is a large inter-patient variability in the *in vivo* plaque material properties. Besides, a large intra-patient variation in YM value was also observed. For two slices from patient P10, YM values in both directions of the stiff plaque were 17.5 times as stiff as those of the soft one.

### Impact of *in vivo* Material Properties on Stress/Strain Calculations

**Figure 5:**
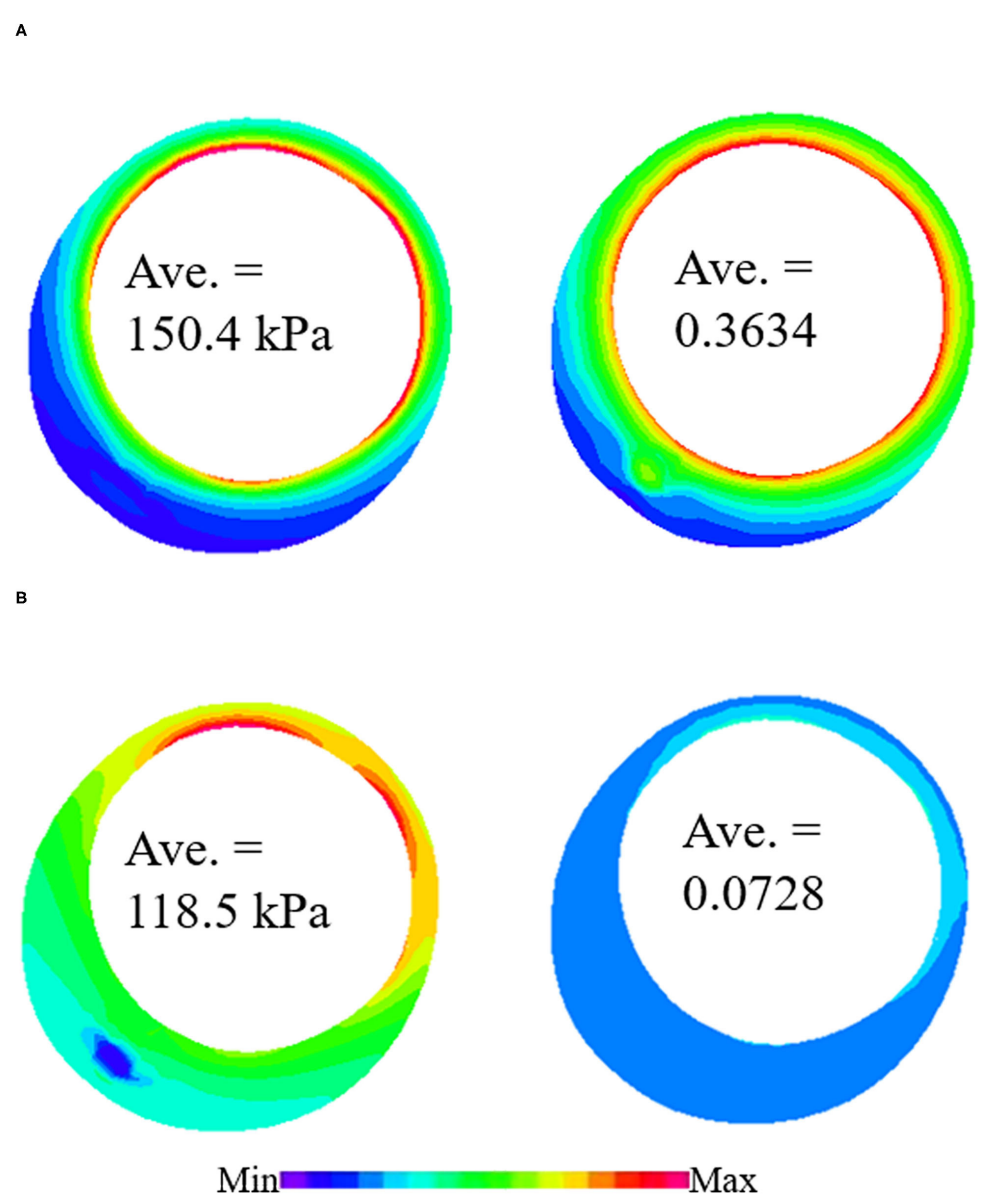
Plaque wall stress/strain (PWS/PWSn) distributions from 3D thin-slice structure-only models with *in vivo* and *ex vivo* materials. **(A)** PWS/PWSn distribution from 3D thin-slice model with in vivo material. **(B)** PWS/PWSn distribution from 3D thin-slice model with in vivo material.

In most published studies, *in vivo* image-based computational models used material properties obtained from *ex vivo* tissue samples, since patient-specific *in vivo* material properties were normally not available. To investigate the influence of this simplification on biomechanical results, 3D thin-slice structure-only models with patient-specific *in vivo* material and *ex vivo* material properties were constructed to simulate stress/strain distributions in each plaque and compare the differences. Figure 5 shows the PWS/PWSn differences on one sample slice using *in vivo* and *ex vivo* materials.

The average values of PWS/PWSn on the luminal surface are given in Table 3. The relative difference between two models was calculated using the following formulas:


(4) $$\begin{array}{l} Relative\;differnce\;in\;PWS\\ \;=\;(PWS_{in\;vivo}-PWS_{ex\;vivo})/PWS_{ex\;vivo} \end{array}$$

and


(5) $$\begin{array}{l} Relative\;differnce\;in\;PWSn\\ \;=\;(PWSn_{in\;vivo}-PWSn_{ex\;vivo})/PWSn_{ex\;vivo} \end{array}$$

**Table 3:** Comparison of average values of plaque wall stress/strain (PWS/PWSn) on luminal surface from thin-slice structure-only models with *in vivo* and *ex vivo* materials.

**Patient/Site**		**PWS (kPa)**		**Relative difference (%)**	**PWSn**		**Relative difference (%)**
		***In vivo*** **material**	***Ex vivo*** **material**		***In vivo*** **material**	***Ex vivo*** **material**	
P1	Site 1	53.5	70.1	−23.65	0.1227	0.0560	118.98
	Site 2	71.5	80.1	−10.78	0.0613	0.0576	6.34
P2	Site 1	77.0	79.7	−3.39	0.0649	0.0625	3.84
P3	Site 1	154.1	123.9	24.31	0.3076	0.0745	312.60
	Site 2	103.5	107.3	−3.50	0.0840	0.0696	20.76
P4	Site 1	86.9	86.3	0.70	0.0620	0.0628	−1.27
	Site 2	139.4	139.4	0.40	0.0866	0.0824	5.10
P5	Site 1	134.9	114.8	17.59	0.2431	0.0722	236.55
P6	Site 1	77.1	89.0	−13.34	0.0913	0.0606	50.61
P7	Site 1	130.8	120.2	8.82	0.0607	0.0718	−15.45
P8	Site 1	150.4	118.5	**26.96**	0.3634	0.0728	398.89
P9	Site 1	53.6	67.6	**−20.71**	0.0774	0.0567	36.51
	Site 2	140.5	138.4	1.50	0.0799	0.0827	−3.43
P10	Site 1	105.0	90.3	16.30	0.3165	0.0596	**431.28**
	Site 2	91.0	86.6	5.00	0.0562	0.0588	−4.48
P11	Site 1	125.0	110.0	13.69	0.0574	0.0686	**−16.40**
	Site 2	154.7	140.4	10.25	0.2041	0.0822	148.14
P12	Site 1	203.8	168.4	21.01	0.2889	0.0936	208.70
P13	Site 1	102.8	109.9	−6.53	0.0880	0.0677	29.90
	Site 2	110.5	114.2	−3.26	0.0891	0.0709	25.60
Average		113.3	107.8	3.07	0.1403	0.0692	99.64

*The minimum and maximum values are in bold*.

Plaque wall stress/strain (stress/strain) from models using *ex vivo* materials were used as baseline values to see the impact of *in vivo* material parameters values on stress/strain calculations. The relative difference in PWS between the two materials varied from −20.71 to 26.96% with an average value of 3.07%, while the relative difference in PWSn is much higher, ranging from −16.4 to 431.28%. The average value is 99.64%. This shows that the *in vivo* material properties have a much greater impact on strain calculation than on stress calculation.

### Softer *in vivo* Plaque Material Properties Lead to Higher PWSn Distributions

To further investigate how material properties influence PWS/PWSn distributions, a correlation analysis between YM values and PWS/PWSn from computational models from the *in vivo* material were performed. Spearman's correlation analysis was performed, since the data do not satisfy the normal distribution according to Shapiro–Wilk test. The results showed that PWSn had a significantly strong negative correlation with YMc (*r* = −0.9368, *p* < 20.0001). This means that softer plaque material properties could lead to higher PWSn distributions. At the same time, there was a non-significant negative correlation between PWS and YMc (*r* = −0.2631, *p* = 0.2611).

### Axial Stretch Has Considerable Impact on Circumferential Shrinkage, Material Parameter Values, and Stress/Strain Calculations

It should be made clear that all the results presented in this article (shrinkage, material parameter values, YMa and YMc values, and stress/strain calculations) are dependent on the imposed axial stretch (5%) we selected. A sensitivity analysis for an axial stretch was performed to demonstrate the impact of axial shrinkage rate on the results. Two representative slices were selected to show the impact: slice 1 (P5, site 1) representing a soft plaque and slice 2 (P13, site 1) representing a stiff plaque. Three-dimensional thin-slice structure-only modes were constructed for the slices with different axial shrinkage (= 3, 5, 7%). Table 4 presents the results, including circumferential shrinkage (denoted as S), parameter values for the Mooney–Rivlin model, YMa and YMc, and PWS/PWSn. The results indicated clearly that smaller axial stretch led to greater slice shrinkage and softer material (smaller YM values). Larger axial stretch gave smaller slice shrinkage and stiffer material (great YM values). Strain results were closely linked to vessel stiffness: softer material gave larger strain while stiffer material gave smaller strain. Stress values were impacted by material stiffness, strain, and plaque morphology and were slightly more complicated.

**Table 4:** Impact of axial shrinkage on circumferential shrinkage (S), YM, and PWS/PWSn of three representative slices.

**Plaque/site**	**Axial shrinkage**	**S (%)**	**C_1_ (kPa)**	**C_2_ (kPa)**	**D_1_ (kPa)**	**K1 (kPa)**	**YMa (kPa)**	**YMc (kPa)**	**PWS (kPa)**	**PWSn**
P12, Site 1 (soft)	3%	17.34	−154.7	13.5	74.2	4.2	110.5	192.2	208.7	0.3240
	5%	15.52	−177.9	15.5	85.3	4.9	127.1	221.0	203.8	0.2889
	7%	13.17	−210.3	18.4	100.9	5.8	150.2	261.3	196.9	0.2466
P13, Site 1 (mild stiff)	3%	5.87	−798.9	69.8	383.2	21.8	570.6	992.6	103.5	0.1111
	5%	3.86	−892.6	78.0	428.1	24.4	637.6	1,109.0	102.8	0.0880
	7%	1.92	−931.5	81.4	446.7	25.5	665.3	1,157.3	129.1	0.0835

With that, it is fair to state that our material property results are dependent on axial stretch rate, which has a large impact on our results. This is, by nature, a lack of data. To avoid this uncertainty and determine more accurate material properties, patient- and vessel-specific axial stretch data are needed.

## Discussion

Mechanical experiments, such as uniaxial/biaxial loading tests and indentation tests, are commonly performed to determine material properties using *ex vivo* cardiovascular tissues. Even though the abundance of experimental data has accumulated from these tests, publications of *in vivo* coronary material properties are scarce, and we may be filling a gap in the current literature. It is desirable to use *in vivo* material properties in computational modeling to have truly patient-specific coronary plaque models. These models are essential for tailored treatment and precision medicine for each individual patient. In this study, IVUS-based 3D thin-slice structure-only models and Cine IVUS data were combined to determine patient-specific *in vivo* plaque material properties for 20 atherosclerotic plaques from 13 patients. This dataset offers first-hand information on *in vivo* material properties of coronary plaques on a relatively large scale for modeling studies and would serve as a base for similar studies. Our results showed that the *in vivo* coronary plaque material properties have large inter-patient and intra-patient variations. The comparative analysis indicated that the *in vivo* material properties have a considerable impact on biomechanical conditions (especially for strain calculations), emphasizing its importance in more accurate strain/stress calculations for cardiovascular disease research.

The eligibility of the 3D thin-slice structure-only modeling approach was validated in previous publications (Huang et al., 2016; Guo et al., 2018). In these studies, the biomechanical stress/strain from 3D thin-slice models was compared with that from full 3D fluid-structure interaction (FSI) models of the curved coronary vessel. A comparison analysis showed that the relative error between two modeling approaches was smaller than 10%, indicating that 3D thin-slice structure-only plaque models could be used as a good approximation to 3D FSI models.

### *In vivo* and *ex vivo* Plaque Material Property Differences and Patient Variations

Our results from the 13 patients showed that there is large inter-patient and intra-patient variability in plaque material properties. The YM value of the stiffest plaque was 21.4 times higher than that of the softest one from the *in vivo* data. Similar observations were also found in *ex vivo* loading experimental studies on plaque tissues in other arteries (Holzapfel et al., 2004; Teng et al., 2014). Our results also showed that there is large intra-patient variability.

The average of YM values for all slices from the 13 patients is 599.5 kPa in the axial direction and 1,042.8 kPa in the circumferential direction. These values are smaller than the data reported in previous studies quantifying atherosclerotic coronary tissues *ex vivo*. The *ex vivo* material we used in this study has YM values of 937.8 and 1,631.2 kPa in the axial and circumferential directions, respectively (Kural et al., 2012). The experimental data from Hoffman et al. showed that the plaque YM in the axial direction and circumferential directions was 1,070 and 1,800 kPa, respectively (Hoffman et al., 2017). An earlier study also reported that the YM value of atherosclerotic coronary was around 1,900 kPa (Akyildiz et al., 2014). This difference in the stiffness of *in vivo* and *ex vivo* plaques implies that *ex vivo* cardiovascular tissues may not exactly represent their mechanical properties *in vivo*.

### Vessel Material Stiffness Has Greater Impact on Strain Calculations

Material property is an essential element for computational modeling. Our results and previous studies have demonstrated that material properties have a great impact on plaque biomechanics (Akyildiz et al., 2014). By comparing stress/strain results using *in vivo* and *ex vivo* material properties, our results indicate that plaque material stiffness has a greater impact on strain calculation. The relative difference in PWSn could be as high as 431.28%, indicating that softer *in vivo* material properties led to much higher strain values, while that of PWS was much lower, with the average value being 3.07%. Softer plaques expand more under a given pressure, while stiff plaque expands less to do that. Plaque stress values across the whole vessel wall from both *in vivo* and *ex vivo* material models are meant to counteract the same blood pressure. Therefore, their values should be relatively close. Of note, these PWS/PWSn values are the average values on the lumen. The influence of pressure on PWS/PWSn calculations may be different from location to location on the lumen and the whole vessel wall.

### Modeling Limitations

It is natural that we would like our research to be as accurate as possible. However, there is a big difference between the *ex vivo* material and *in vivo* data based on IVUS. Ideally, those material parameters are related to the strain energy of different biological structures that can be modeled in different manners and in an uncorrelated fashion. If we had more data points (such as those obtained from *ex vivo* biaxial mechanical testing), we could try to find the proper models with more parameters quantified. The reality is, with our *in vivo* data, we only have two data points to work with (minimum and maximum lumen circumferences corresponding to diastolic and systolic pressures). Therefore, only two items could be determined. Since we have to find the no-load state of the vessel using our pre-shrink process, only one value could be determined for the chosen material model. The Mooney–Rivlin model was used, since it was found to have a good fit to the *ex vivo* biaxial mechanical testing data we used earlier (Kural et al., 2012). We assumed that those parameters in the Mooney–Rivlin model to be proportionally related to “k” to have one value that can be determined by our *in vivo* data. This is a “one-point” guess to these parameter values. Any more accurate guesses would not be supported by the *in vivo* data. However, people could choose another one value to quantify, such as just c_1_ and c_2_. People can argue equally to support those choices or be against them as well.

One major limitation of the study is the lack of *in vivo* on-site blood pressure data, which have to be obtained using both pressure guidewire and IVUS catheters invasively. Those procedures involve additional risk and cost, and are not done in regular clinical practice. Therefore, it is difficult to acquire both the IVUS image and on-site blood pressure data (like from FFR) from the same patient as the case in this study. In particular, the data set ZhongDa hospital provided included IVUS and Cine IVUS, but no FFR. Noninvasively measured arm cuff pressure was used as a surrogate for intracoronary pressure for all the patients in this study.

Another limitation for the *in vivo* data is that the axial shrinkage was assumed to be 5%. The small axial shrinkage is justified by the fact that vessels with plaques are much stiffer than healthy ones. The actual axial shrinkage could not be determined unless we have the vessel samples, which are, in general, not possible for coronary studies (carotid could have plaques removed to have both *in vivo* and *ex vivo* data to determine axial shrinkage). Clearly, a lot more needs to be done to move forward for more accurate quantification of patient- and location-specific axial shrinkage using *in vivo* data.

*In vivo* coronary vessel material properties are extremely hard to obtain. This study represents our effort in obtaining some first guess using data available to us. Data using dual-catheter can provide both vessel image and on-site blood pressure and allow us to fit material models with more parameter values determined.

Some other limitations of our modeling techniques include: (a) axial movement of IVUS transducer was not considered when acquiring Cine IVUS images (Arbab-Zadeh et al., 1999). The Cine IVUS data are what the transducer could see. The position of the transducer was fixed by pausing the pullout when Cine was taken. A far more severe limitation was not knowing vessel-specific axial stretch (vessel is stretched *in vivo*). Further efforts will be made to ensure a more accurate estimation of *in vivo* plaque material properties (Maso Talou et al., 2016); (b) Our models did not consider the adventitial layer of the coronary vessel, as the IVUS data did not provide it (Mintz et al., 2001); (c) 3D thin-slice models instead of full 3D models with curvatures were used, since we only have Cine data for the given slices. Have we used full 3D vessel models, we would also have issues that Cine data could not support the material properties for other locations; (d) residual stress was not included, as no patient-specific opening angle data were available (Fung and Liu, 1992; Ohayon et al., 2007).

### Model Validation

It should be stated and understood that vessel material properties and subsequent stress/strain calculations are associated with model assumptions made in this study and are subjected to several limitations given above. Ultimate validation of material properties should come from mechanical testing data using real tissue samples that are normally not available for coronary studies. However, our approach has a “self-validation” nature: since we matched *in vivo* vessel Cine data, our stress/strain calculations using the same model assumptions are supported by the matched vessel deformations. This is advancement for patient-specific coronary models over those from the available literature that only used material parameters.

## Data Availability Statement

The original contributions presented in the study are included in the article/supplementary material, further inquiries can be directed to the corresponding author/s.

## Ethics Statement

The studies involving human participants were reviewed and approved by Zhongda Hospital, Southeast University (Nanjing, China). The patients/participants provided their written informed consent to participate in this study.

## Author Contributions

DT, GM, JZ, and GSM: conceived and designed the experiments. JZ, YQ, XZ, LC, and GM: collected the data. LW, RL, XG, and KB: analyzed the data. LW and DT: drafted the manuscript. AM, XG, GSM, and DT: revised the manuscript. All the authors read, edited, and approved the final version of the manuscript.

## Funding

This research was supported in part by the National Natural Science Foundation of China (Grant Nos.: 11672001, 11972117, and 11802060), the Natural Science Foundation of Jiangsu Province (Grant No.: BK20180352) and Jiangsu Province Science and Technology Agency (Grant No.: BE2016785), the Fundamental Research Funds for the Central Universities, and Zhishan Young Scholars Fund (Southeast University, No.: 2242021R41123).

## Conflict of Interest

The authors declare that the research was conducted in the absence of any commercial or financial relationships that could be construed as a potential conflict of interest.

## Publisher's Note

All claims expressed in this article are solely those of the authors and do not necessarily represent those of their affiliated organizations, or those of the publisher, the editors and the reviewers. Any product that may be evaluated in this article, or claim that may be made by its manufacturer, is not guaranteed or endorsed by the publisher.
